# Photocatalytic Dinitrogen Reduction to Ammonia over Biomimetic FeMoS_*x*_ Nanosheets

**DOI:** 10.1021/acsomega.4c03076

**Published:** 2024-04-24

**Authors:** Suresh Thangudu, Chein Hou Wu, Kuo Chu Hwang

**Affiliations:** † Department of Chemistry, National Tsing Hua University, Hsinchu 30013, Taiwan R.O.C; ‡ Department of Biomedical Engineering and Environmental Science, National Tsing Hua University, Hsinchu 30013, Taiwan R.O.C

## Abstract

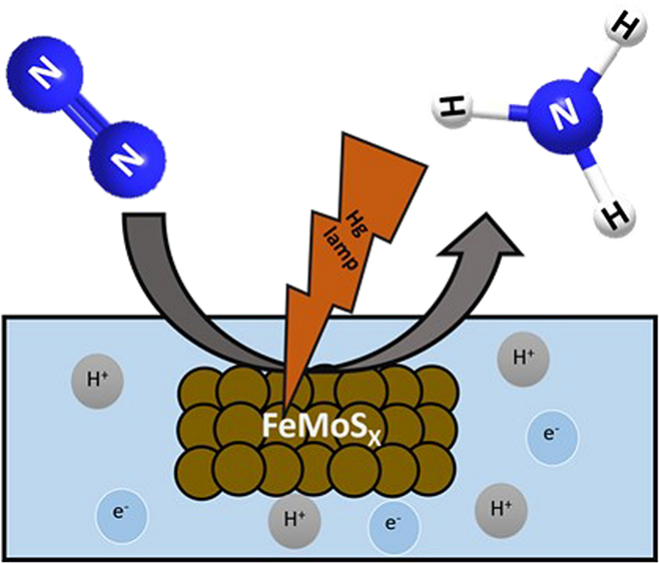

Reduction of atmospheric dinitrogen (N_2_) to ammonia (NH_3_) using water and sunlight in the absence of sacrificial reducing reagents at room temperature is very challenging and is considered an eco-friendly approach to meet the rapidly increasing demand for nitrogen storage, fertilizers, and a sustainable society. Currently, ammonia production via the energy-intensive Haber–Bosch process causes ∼350 million tons of carbon dioxide (CO_2_) emission per year. Interestingly, natural N_2_ fixation by the nitrogenase enzyme occurs under ambient conditions. Unfortunately, N_2_ fixation on biomimetic catalysts has rarely been studied. To mimic biological nitrogen fixation, herein, we synthesized the novel iron molybdenum sulfide (FeMoS_*x*_) micro-/nanosheets via a simple hydrothermal approach for the first time. Further, we successfully demonstrated the photochemical conversion of N_2_ to NH_3_ over a biomimetic FeMoS*_x_* photocatalyst. The estimated yield is around 99.79 ± 6.0 μmol/h/g photocatalyst with a quantum efficiency of ∼0.028% at 532 nm visible-light wavelength. Besides, we also systematically studied the influence of key factors to further improve NH_3_ yields. Overall, this study paves a new pathway to fabricate carbon-free, photochemical N_2_ fixation materials for future applications.

## 1 Introduction

Artificial nitrogen (N_2_) fixation for the synthesis of ammonia (NH_3_) has received significant attention in the 21st century to replace the conventional Haber–Bosch process, where the reaction was carried out under high-energy-demanding conditions (15–25 MPa, 300–550 °C) in the presence of a metallic iron catalyst.^1,2^ In the Haber–Bosch process, natural gas is mainly used as the hydrogen source, which typically produces ∼1.87 tons of CO_2_ emissions per ton of NH_3_ production.^3^ In view of global climate change, a novel process is required to fix N_2_ to NH_3_ in a less energy-demanding pathway. In contrast, due to its very strong N≡N triple bond, N_2_ cleavage and functionalization at ambient conditions have been challenging tasks for years. The rate-determining step in the synthesis of NH_3_ is primarily the cleavage of the very strong N≡N bond, which renders the reduction of N_2_ very difficult.^4^

In nature, the amount of N_2_ being converted to NH_3_ and its derivatives in plants is on the order of ∼10^8^ tons per year.^3^ The process of N_2_ binding and activation involves the nitrogenase enzyme, which contains iron–molybdenum–sulfur constituents as key components, which are further called the iron molybdenum cofactor (FeMoco).^5,6^ However, the exact N_2_ fixation mechanism in nature remains unclear. Inspired by the role of FeMoco, several groups have actively synthesized various transition-metal complexes and examined N_2_ reduction to NH_3_.^7−17^ However, these systems are very different from the conventional Haber–Bosch process, where strong reducing agents and organic solvents were utilized with poor conversion efficiencies. Hence, several efforts have been made to conquer N_2_ reduction by various approaches such as thermal,^18−20^ photochemical,^21−24^ and electrochemical.^25−33^ Despite the tunable and scalable nature of electrocatalysis, hydrogen evolution reaction (HER) competition, low NH_3_ yields, and low electrocatalytic efficiencies still impede practical implementation.^34^ The low NH_3_ yield of electrocatalysts also makes it difficult to measure the amount of NH_3_ generated by the electrochemical nitrogen reduction reaction (e-NRR) accurately, which affects its credibility and reproducibility. In addition, NH_3_ contamination from outside systems can have a significant effect on the accuracy of the measurements of NH_3_ yields from electrocatalysts.^34^ Currently, photochemical approaches are very promising since they use semiconductors as catalysts, water as the universal solvent, and solar light as a driving force to produce chemical reactions. However, due to the poor interfacial charge transfer in the semiconductor photocatalytic process, N_2_ reduction efficiencies are quite far from satisfactory.^35,36^ To this end, coupling photochemistry with biomimetic catalysts is fascinating to accelerate N_2_ reduction to NH_3_ in a green manner. As a result, biomimetic chalcogels such as FeMoS-Sn_2_S_6_^37^ and Mo_2_Fe_6_S_8_(SPh)_3_^38^ were explored for N_2_ reduction with excellent outcomes. Despite having stability issues and lower efficiencies (<0.02%), they have a significant impact on the N_2_ activation community for developing a new biomimetic strategy. Thereafter, nitrogenase-CdS biohybrids were utilized for N_2_ photofixation, which offers a decent quantum efficiency (3.3%). However, the utilization of expensive proteins as well as the degradation of the catalyst during catalytic reaction makes it far from satisfactory.^39^ Recently, our group reported that biomimetic constituents such as Fe- and Mo-incorporated mesoporous silica (FMZ-0.05) catalysts can reduce N_2_ to NH_3_ efficiently with greater quantum efficiencies (0.92% at 532 nm wavelength).^40^ Overall, it is an intriguing challenge to find suitable biomimetic photocatalysts to fix N_2_ to NH_3_ with lower energy demands under ambient conditions.

**Scheme 1:**
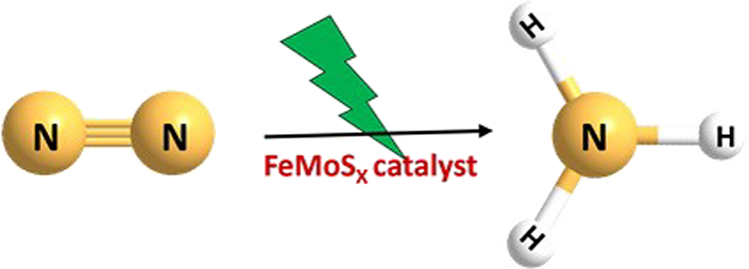
Schematic Representation for Photoinduced Dinitrogen Reduction to Ammonia Using FeMoS_*x*_ Sheets as a Catalyst

Inspired by the role of the nitrogenase enzyme and its constituents in biological nitrogen fixation, here, we synthesized a novel biomimetic iron molybdenum sulfide (FeMoS_*x*_) catalyst via a simple hydrothermal method for the first time. This biomimetic catalyst was examined for the conversion of N_2_ to NH_3_ under light, as shown in Scheme 1. Moreover, we experimentally demonstrated the influence of key factors to achieve efficient N_2_ reduction. To the best of our knowledge, this work represents the first demonstration of the fabrication of a FeMoS_*x*_ catalyst for photoinduced dinitrogen reduction to ammonia via a biomimetic FeMoS_*x*_ photocatalyst, which produced ∼99.79 ± 6.0 μmol/h/g catalyst, with an appreciable quantum efficiency of 0.028% (532 nm, 43.5 mW/cm^2^) in the visible region.

## 2 Results and Discussion

**Figure 1:**
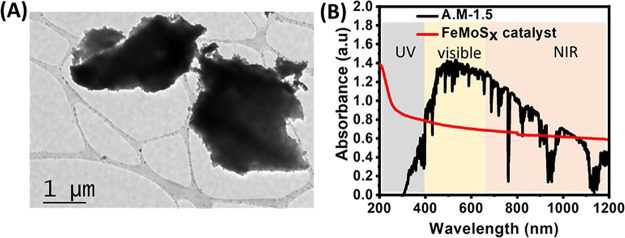
(A) Transmission electron microscopy (TEM) image of the FMS catalyst. (B) UV–vis–NIR extinction spectra of the FMS catalyst and comparison to the AM 1.5 solar spectrum (adapted from ref (41) with permission from the American Chemical Society).

First, structural morphology and particle size of as-synthesized FeMoS*_x_* micro-/nanosheets were confirmed by scanning electron microscopy (SEM), which reveals a sheet-type morphology with an average particle size of around ∼2 μm (Figure S1). Subsequently, transmission electron microscopy (TEM) analysis was performed, which confirmed the sheet-type morphology of FeMoS_*x*_ as well (Figures 1A and S2). The resulting FeMoS_*x*_ sheets were labeled as FMS. The UV–vis–NIR absorption spectrum of FMS exhibits a broad optical absorption throughout the solar spectral range (Figure 1B). Subsequently, the Brunauer–Emmett–Teller (BET) method was performed to measure the average surface area of the FMS catalyst, and the estimated surface area is 3.6 m^2^/g (Figure S3). Powder X-ray diffraction (PXRD) analysis reveals the amorphous nature of the FMS sheets (Figure S4). Besides XRD, high-resolution TEM and selected area electron diffraction (SAED) analysis also reveal no clear lattice on FMS sheets, indicating their amorphous nature (Figure S5). The coexistence of elements in FMS was confirmed by energy-dispersive X-ray analysis (EDX), which reflects the presence of Fe, Mo, and S elements (Figure S6).

**Figure 2:**
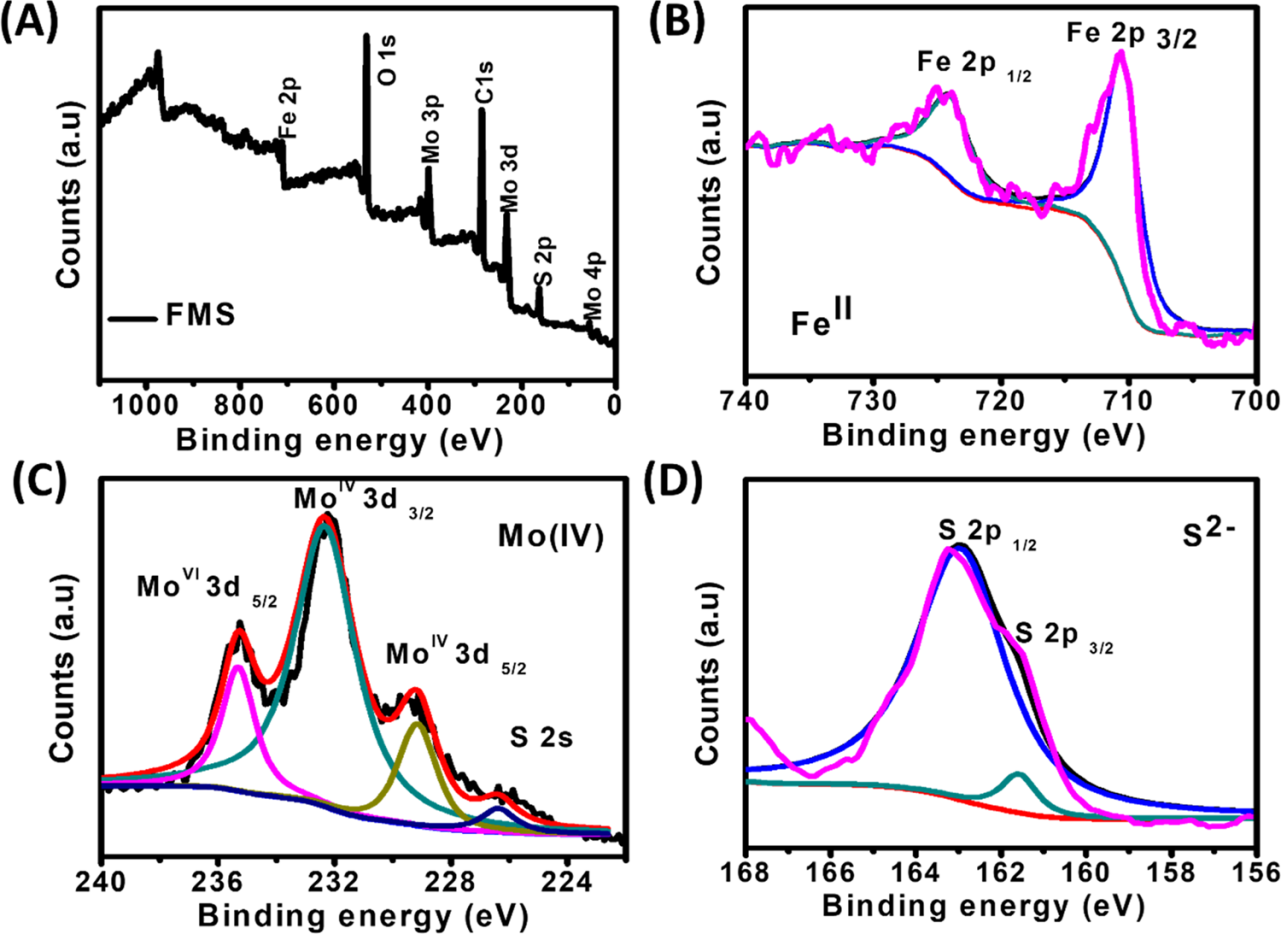
(A) XPS survey spectrum and (B–D) multiplex scans of Fe 2p, Mo 3d, and S 2p, respectively.

Thereafter, oxidation states and the chemical environment of the FMS catalyst were confirmed by using X-ray photoelectron spectroscopy (XPS). The XPS survey spectrum in Figure 2A reveals the existence of Fe, Mo, and S elements. The XPS scan of Fe in Figure 2B shows two characteristic peaks at 710.5 and 724.1 eV for Fe 2p_3/2_ and Fe 2p_1/2_, respectively, which indicate the presence of the Fe(II) oxidation state. Similarly, the XPS scan for Mo exhibited two major characteristic peaks at 229.05 and 232.1 eV corresponding to Mo(IV) 3d_5/2_ and Mo(IV) 3d_3/2_, respectively. In addition to the Mo(IV) 3d_5/2_ signal, there also exists another peak at a slightly higher binding energy region corresponding to Mo(VI) 3d_5/2_ with a +6 oxidation state, which is presumably due to the incomplete reduction of [MoS_4_]^2–^ during the hydrothermal process (Figure 2C). As shown in Figure 2D, the sulfur (S) 2p XPS scan clearly shows the doublet corresponding to S 2p_3/2_ at 161.6 eV binding energy, revealing the −2 oxidation state of sulfur, which is consistent with previous reports.^42,43^ The appearance of a shoulder peak at higher binding energies in the S 2p region indicates the higher oxidation state of Mo due to incomplete reduction. Chemisorbed oxygen spectra are shown in Figure S7.

**Figure 3:**
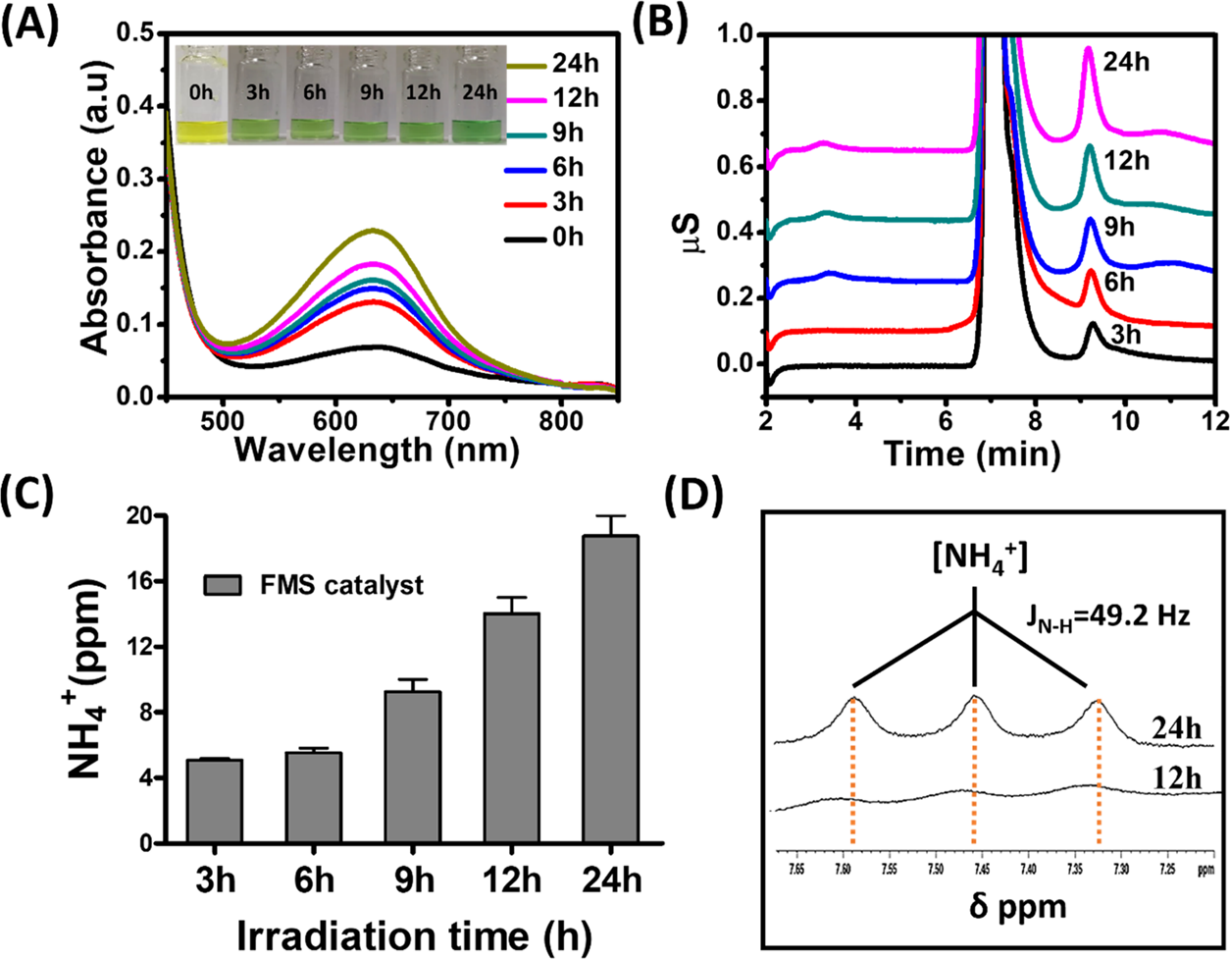
(A) UV–vis absorption spectra of the indophenol assay (inset images show a gradual enhancement of the indophenol complex). (B) Ion chromatogram spectra of aliquots obtained during fixation at different time points. (C) Kinetic plot of ammonia evolution plotted as a function of photoirradiation. (D) ^1^H NMR spectra of the generated [NH_4_]^+^ at different time intervals.

To mimic the biological process of nitrogen fixation, synthesized FMS catalysts were used for N_2_ photofixation to NH_3_. All of the photochemical experiments were conducted at room temperature using a 100 W high-pressure Hg lamp as a light source. The desired quantity of the catalyst was placed in an aqueous solution in the presence of a proton source (pyridinium chloride) and a sacrificial electron donor (sodium ascorbate) and subsequently illuminated with light (350 nm, light intensity on sample: 200 mW/cm^2^) under a N_2_ atmosphere at room temperature. The reaction mixture was monitored for a period of 24 h under constant stirring. The photochemical setup for dinitrogen reduction is shown in Figure S8. Aliquots of samples were collected from the reaction solution at different time points, and the amount of ammonium ([NH_4_^+^]) ions was determined using the qualitative indophenol method.^44^ Further quantitative measurement of the ammonium ion was measured by the direct ion-exchange chromatographic method without any sample pretreatment (see the Methods section and the Supporting Information for details). The resulting [NH_4_^+^] concentration was accurately estimated with the help of calibration curves (Figure S9), obtained from standard concentrations of [NH_4_^+^] (0.1–2 ppm).^40^ During the photoirradiation process, the reaction samples were collected at various time points and assayed for the presence of NH_3_. As shown in Figure 3A, the absorbance intensity at 630 nm of the indophenol complex increases as a function of time, which presumably is related to the increase in the concentration of ammonium ions (optical images of the indophenol assay are shown in the inset; the intensity of color changes over time). Also, the ion chromatograms in Figure 3B quantitatively confirm that the generation of [NH_4_]^+^ increases linearly as a function of illumination time. A kinetic plot of NH_3_ generation (in parts per million (ppm)) over the irradiation time is shown in Figure 3C. To further assist with the generation of NH_3_, we also carried out ^1^H NMR analysis. As shown in Figure 3D, a sharp triplet corresponding to NH_3_ was observed with a *J*_N–H_ coupling constant of 49.2 MHz (^1^H NMR full spectrum shown in Figure S10). In the present photocatalytic system, the estimated yield of ammonia on the FMS catalyst is to be 64.0 ± 5.0 μmol/h per 1 gram of the FMS catalyst.

**Figure 4:**
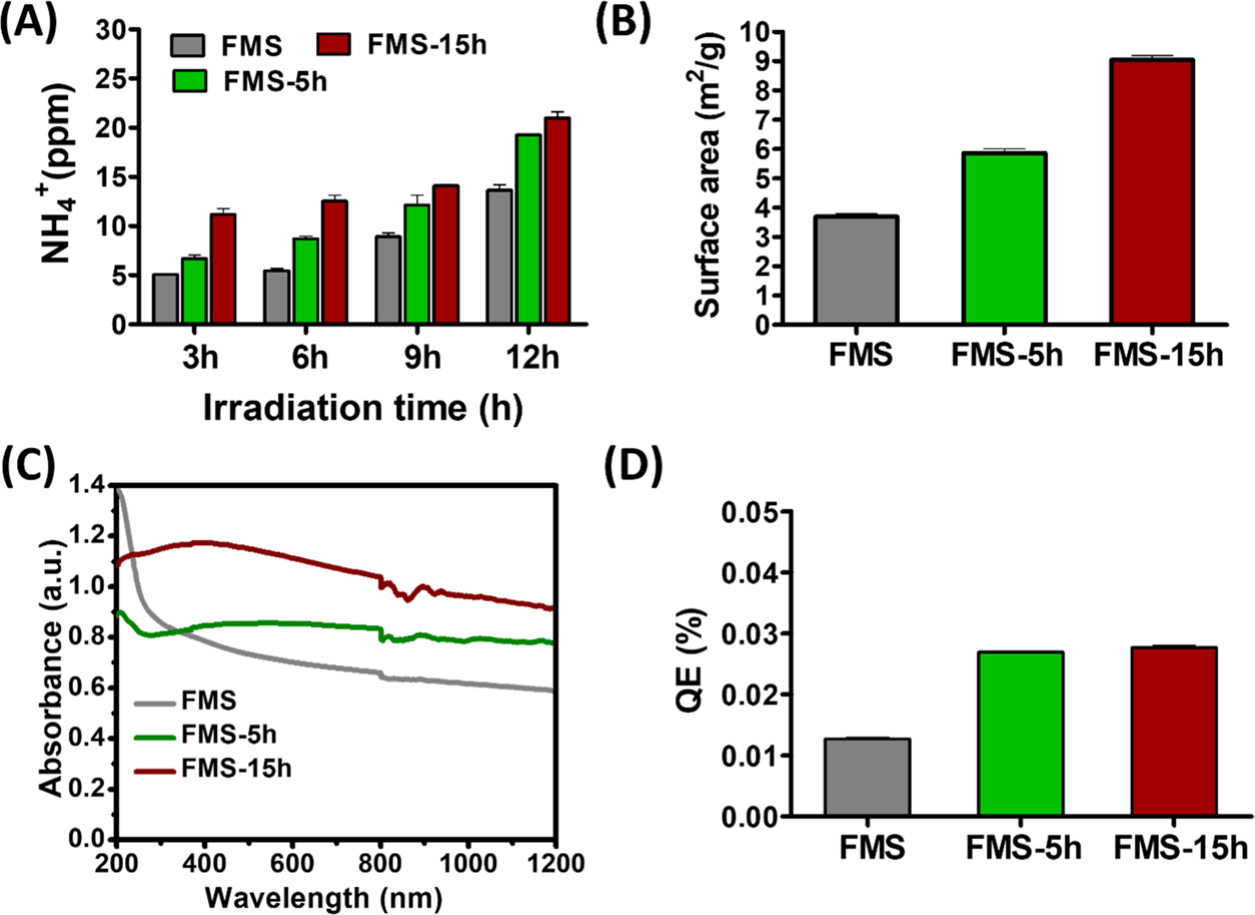
(A) Yield of ammonia on various synthesized catalysts. (B) Surface area of the catalyst. (C) Absorption spectra of the catalyst. (D) Quantum efficiencies on various catalysts at 532 nm visible light.

Furthermore, we carried out photocatalytic N_2_ reduction by using smaller-sized FeMoS*_x_* nanosheets, which were prepared via the tip sonication method (see the Section 4). As-obtained catalysts are labeled as FMS-5h (5h tip-sonicated FMS microsheets) and FMS-15h (15h tip-sonicated FMS microsheets). As expected, the yields of ammonia were gradually increased as follows: 64.0, 94.90, and 99.79 μmol/h per 1 g of catalyst on FMS, FMS-5h, and FMS-15h catalysts, respectively. Figure 4A shows the time-dependent formation of ammonia on the synthesized catalysts (indophenol and ion chromatograms are shown in Figures S11 and S12). We hypothesized and confirmed that the reason behind the enhancement of ammonia is mainly due to the surface area and optical absorption of the catalyst. Figure 4B clearly shows an increase in the surface area of the catalyst (as follows 3.6, 5.7, and 8.9 m^2^/g for FMS, FMS-5h, and FMS-15h, respectively) while decreasing the particle size. Besides surface area, we also noticed that the optical absorption of the catalyst was also enhanced in the visible region compared to the FMS catalyst (Figure 4C), which is believed to be a key factor in the present N_2_ reduction system. Further, quantum efficiency experiments were conducted at the 532 nm visible light wavelength region (output power intensity on sample is 43.5 mW/cm^2^) on various synthesized catalysts. As shown in Figure 4D, the estimated QEs are as follows: 0.013, 0.027, and 0.028 on FMS, FMS-5h, and FMS-15h catalysts, respectively. From our findings, we anticipated that the surface area of the catalyst and light absorption of the catalyst would improve the yields. Several control experiments were performed to prove that the present N_2_ reduction is mainly achieved on the FeMoS*_x_* photocatalyst. For instance, no significant amount of ammonia generation was observed in the absence of light, a proton source, or a catalyst (Figure S13). To further confirm the source of N in NH_3_, instead of N_2_ gas, we carried out N_2_ photofixation under an argon (Ar) atmosphere, which resulted in no notable formation of NH_3_ detected under argon (Figure S14). Besides, isotope labeling experiments were carried out to further assist the N source in NH_3_. Instead of ^14^N_2_ gas, ^15^N_2_ isotope labeled gas was utilized for photocatalytic N_2_ fixation on the FMS catalyst. As shown in Figure S15, a sharp doublet was observed in ^1^H NMR corresponding to the ^15^NH_4_^+^ ion with a *J*_N–H_ coupling constant of 72 Hz, which is in agreement with the reported literature.^40^ These results strongly confirm that nitrogen gas is indeed the major source of ammonium nitrogen. The durability of the FeMoS*_x_* catalyst shows that there is no loss of photoreactivity observed up to three times of the cycling test, indicating that the catalyst was photostable during photocatalytic nitrogen fixation and recyclable (Figure S16). Furthermore, TEM analysis of the FMS catalyst after the catalytic reactions reveals that the morphology of the FMS catalyst remains the same as sheets even after long photocatalytic reactions (Figure S17). Similarly, XPS analysis also indicated no structural deformation of the catalyst after a long photocatalytic reaction time (Figure S18). Noticeably, gradual decay was observed after three cycles of photocatalytic reaction. The possible reasons for the lower recyclability on the present catalyst are as follows: (i) the photocatalyst particles may be lost throughout each collection and rinse cycle and (ii) the masking of the active site on the catalyst by intermediate/ammonia molecules on the surface of the catalyst. According to the control experiments, here, we propose a plausible mechanistic pathway shown in Figure S19. It is well-known that transition metals are likely to act as an efficient N_2_ activator to weaken the N≡N bond via π back-bonding phenomena.^45^ Moreover, the FeMo cofactor plays a vital role in nitrogenase in natural nitrogen fixation to weaken the N≡N bond, but the exact mechanism remains unknown. On this basis, we hypothesized that the N≡N bond will weaken on the present biomimetic catalyst (either the Fe or Mo site), followed by cleavage of the weakened N≡N bond by photoexcited electrons on the FMS catalyst. Further, the activated N_2_^–^ species react with the absorbed hydrogen protons on the surface of the catalyst to form ammonia via a hydrazine intermediate. Notably, we observed a very tiny amount of hydrazine as a byproduct during the photofixation process (Figure S20). It is worth mentioning that the proton source plays a very crucial role in the reductive conversion of N_2_ to achieve higher yields of ammonia. We have examined different proton sources, such as methane, hydrogen gas, and water, but all of these have poor conversion yields as compared to pyridinium chloride in our system (Figure S21). Besides, sacrificial electron donors are also needed to enhance the N_2_ reduction by minimizing the charge recombination of photoexcited electrons. Even in natural systems, the nitrogenase enzyme utilizes sacrificial electron donors such as ferredoxin or flavodoxin during the fixation of N_2_ to NH_3_.

The light-harvesting properties of micro-/nanomaterials are highly tunable. Moreover, the catalytic performance of the nanosheets will be further enhanced by light irradiation. To better understand the effect of photoirradiation on the present FMS system, we performed N_2_ fixation experiments under dark and light conditions at identical conditions. When compared to the N_2_ fixation in the dark, N_2_ reduction under light produces 14-fold higher yields of ammonia, which reflects the photo-favorableness of the present approach (Figure S22). To further determine the efficacy of the present FMS catalyst, we synthesized and examined several photocatalysts for the reduction of N_2_ to NH_3_. Among all of them, the biomimetic FeMoS*_x_* photocatalyst shows the best efficiency for the conversion of N_2_ to NH_3_ at room temperature (Table S1). From our results, we hypothesized that the biomimetic nature of the FeMoS*_x_* catalyst (like the FeMo cofactor, but the exact mechanism is unknown) plays a key role in the successful conversion of N_2_ to NH_3_ at room temperature with appreciable quantum efficiencies. To the best of our knowledge, the present method facilitates and provides a deep insight into achieving an efficient N_2_ reduction to NH_3_ by using biomimetic nano-/microsheets. The present efficiency for N_2_ reduction on the FeMoS*_x_* photocatalyst makes it more favorable than the reported biomimetic photocatalysts (Table S2). As expected, the overall efficacy of N_2_ reduction to ammonia is still far from that of the reported photocatalysts (Table S3). However, in our findings, we noticed that some key factors, such as (i) Fe–Mo cofactor, (ii) surface area of the catalyst, and (iii) light absorption of the catalyst, greatly influence the ammonia formation yields and should be considered in future N_2_ photofixation studies.

## 3 Conclusions

In summary, we have successfully synthesized the novel FeMoS*_x_* catalyst by a simple hydrothermal route for the first time. The present catalyst can mimic the N_2_ fixation in nature and produce NH_3_ (99.79 ± 6.0 μmol/h/g catalyst) in a fully solar-driven process under ambient conditions. The external quantum efficiency of this biomimetic nitrogen reduction reached 0.028% in the visible-light region (532 nm wavelength) for these complicated six electrons’ reduction process. Furthermore, we discovered that the present efficiency is still not limited and that we can improve it by increasing the surface area and light absorption of the catalyst to meet real-time needs. We envision that this study might open a new pathway with deep scientific insights for fabricating highly efficient photocatalysts for the efficient fixation of photochemical N_2_ to NH_3_.

## 4 Experimental Section

### 4.1 Synthesis of FeMoS*_x_* Microsheets

All reagent chemicals were purchased from Sigma-Aldrich without further purification. Sodium tetrathiomolybdate ((NH_4_)_2_MoS_4_, 99.97%, Sigma-Aldrich) and iron(II)sulfate heptahydrate (FeSO_4_·7H_2_O, ACS reagent, ≥99.0%, Sigma-Aldrich) are mixed in the molar ratio of 1:2. Appropriate amounts of polyvinylpyrrolidine (PVP, average mol wt 40 000, Sigma-Aldrich) and sodium acetate (NaAc, ACS reagent, ≥99.0%, Sigma-Aldrich) were dissolved in a 40 mL mixed solution of metal precursors (25 mL of H_2_O and 15 mL of glycol) at a constant speed under stirring at room temperature for 10 min. After that, the mixed solution was transferred into an autoclave in an electric oven at 200 °C for a period of 16 h and then the autoclave was cooled to room temperature naturally. The obtained product was centrifuged and washed several times with deionized water and ethanol before vacuum-drying at 80 °C.

### 4.2 Synthesis of FMS-5h and FMS-15h Nanosheets

The above-synthesized FeMoS*_x_* microsheets were dispersed in water and tip-sonicated around 5 and 15 h to achieve FMS-5 h (5 h tip-sonicated FeMoS*_x_* sheets) and FMS-15h catalysts (15 h tip-sonicated FeMoS*_x_* sheets), respectively.

### 4.3 Photochemical Reaction

All photocatalytic activity experiments were carried out at room temperature under a high-pressure 100 W Hg lamp as a light source. Ammonia evolution experiments were carried out in sealed reaction vials, and 1 mg/mL catalyst was added into an aqueous solution containing pyridinium chloride (50 mM) and sodium ascorbate (5 mM). After subsequent illumination with a 100 W high-pressure Hg lamp, the power intensity at the reaction surface was ∼200 mW/cm^2^. Aliquots of the reaction were collected at different time intervals and assayed for ammonia by using the indophenol method and ion chromatography (cation-exchange column). The external quantum efficiency was calculated by using the following formula: Quantum efficiency = (100% × number of generated ammonia × 6)/number of incident photons. For all of the photocatalytic experimental studies, we have used nitrogen gas with 5.0 grade (99.999%) purity directly without further purification. For ^15^N experiments, nitrogen-^15^N_2_ (98 atom % ^15^N) gas was used directly without further purification. Precisely, a nitrogen-^15^N_2_ labeling experiment was performed by using anhydrous solvents and reactions under the standard condition (instead of N_2_ gas, 98% pure nitrogen-^15^N_2_ gas was filled in the reaction system).

### 4.4 Qualitative Analysis of Ammonia by the Indophenol Assay

In the indophenol method, 0.5 mL of reaction aliquots was treated with 0.1 mL of a phenolic solution in 95% ethanol/water (1.2 g of phenol in 10 mL of 95% ethanol/water), 0.1 mL of 0.5% Na[Fe(CN)_5_NO] solution, and 0.375 mL of alkaline sodium citrate solution (2 g of sodium citrate and 0.1 g of NaOH in 10 mL of water mixed with 2.5 mL of 5% NaClO solution). Reaction mixtures were kept at room temperature to develop the color before spectrophotometric testing. Spectrophotometric analysis was performed in a spectrophotometer, with λ_max_ observed at 630 nm corresponding to the indophenol complex. To confirm that the observed formation of ammonia is truely due to the action of FeMoS_*x*_ catalyst, several control experiments were conducted with the absence catalyst, nitrogen gas, proton source, showed no ammonia formation.

### 4.5 Quantitaive Determination of Ammonia by Ion Chromatography

0.5 mL aliquots of illuminated reaction solutions were taken at different time intervals, diluted 10 times with deionized water, and assayed for ammonia using the ion-chromatographic method.3 Ammonia quantification was conducted in a DX-120 instrument using an IonPac CS16 and the capillary guard column, separately. 20 mM methanesulfonic acid (MSA) was used as the eluent, and a flow rate of 1 mL/min was maintained throughout the sample runs. Different standard concentrations of ammonium were prepared in the range of 0.1–2 ppm (Figure S3a). Calibration curves were obtained by plotting the peak area (in μS) vs concentration of the standard (in ppm). Quantities of ammonium of reaction aliquots were estimated from the peak area for the respective cation.

### 4.6 Determination of Hydrazine Hydrate

The hydrazine present in the solution was determined by the method of Watt and Chrisp (S1). The calibration curve was plotted as follows. A series of standard solutions were prepared by pipetting suitable volumes of the hydrazine hydrate in colorimetric tubes and making up to 5 mL. Then, 5 mL of color reagent (a mixture of para-(dimethylamino) benzaldehyde (5.99 g), HCI (concentrated, 30 mL), and ethanol (300 mL) was used as a color reagent) was added and stirred for 10 min at room temperature. Thereafter, the absorbance of the resulting solution was measured at 460 nm, and the yields of hydrazine were estimated from a standard curve.

## Supporting Information Available

Additional characterization methods (SEM, XRD, HRTEM, EDX, and XPS oxygen spectra); ammonia detection methods; N_2_ fixation control experiments; N_2_ fixation under different photocatalysts; NMR data; possible mechanisms; and literature comparison (PDF)
