# Supplementary material for: Photocatalytic Dinitrogen Reduction to Ammonia over Biomimetic FeMoSx Nanosheets
Source: ACS Omega. 2024 Apr 24;9(18):20629–35. doi: 10.1021/acsomega.4c03076 (PMC11080007; doi:10.1021/acsomega.4c03076)
Supplement: Supplementary file 1 — ao4c03076_si_001.pdf [file ao4c03076_si_001.pdf]

**Supporting Information**

# Photocatalytic Dinitrogen Reduction to Ammonia over a Biomimetic $FeMoS_x$ Nanosheets

*Suresh Thangudu* [a#], *Chein Hou Wu* [b] *and Kuo Chu Hwang* [a*]

[a] Department of Chemistry, National Tsing Hua University, Hsinchu, Taiwan R.O.C.

[b] Department of Biomedical Engineering and Environmental Science, National Tsing Hua University, Hsinchu, Taiwan R.O.C.

#. Current address: Department of Radiology, Stanford University, USA.

Corresponding author Email: kchwang@mx.nthu.edu.tw.

KEYWORDS. Nanomaterials, biomimetic, nitrogen fixation, photo fixation, ammonia, influence factors

# Table of contents

## S1. Supplementary figures

**Figure S1**. Figure S1. SEM images of FMS catalyst.-------------------------------------------------4

**Figure S2**. Figure S2. High and low resolution HRTEM images of FMS catalyst. ----------------4

**Figure S3**. BET surface area analysis report.-------------------------------------------------------------5

**Figure S4**. XRD spectra of FeMoSX microparticles .-------------------------------------------------5

**Figure S5**. SAED pattern of FeMoSX particles--------------------------------------------------------6

**Figure 6**. EDX Elemental composition analysis of FeMoSX microparticles. -----------------------6

**Figure S7**. High reosolution XPS spectra of O 1s.-----------------------------------------------------7

**Figure S8**. Reaction setup photograph of photochemical nitrogen reduction reaction.-------------7

**Figure S9**. standard calibration curves. (a) calibration curve for Indophenol assay. (b) calibration curve for Ion chromatogram.---------------------------------------------------------------------------8

**Figure S10**. 1H NMR spectra. $^{1}H$ NMR spectra of photo catalytic reduction of dinitrogen to ammonia at standard experimental condition. Conditions : DMSO-d6, 300 MHz. 1 JN-H = 49.2 Hz. -----------------------------------------------------------------------------------------------8

**Figure S11**. Photocatalytic dinitrogen reduction by using FMS-5h catalyst. a) Indophenol qualitative assay. b) Ion-chromatogram quantitative assay. c) kinetic plot of ammonia generation during the photoirradiation.-----------------------------------------------------------------------------9

**Figure S12**. Photocatalytic dinitrogen reduction by using FMS-15h catalyst. a) Indophenol qualitative assay. b) Ion-chromatogram quantitative assay. c) kinetic plot of ammonia generation during the photoirradiation. d) SEM image of the FMS-15h catalyst.--------------------------------10

**Figure S13**. Representative UV-Vis of indophenol assays of 12h aliquots obtained during photocatalytic dinitrogen reduction at different conditions.------------------------------------------11

**Figure S14**. Photocatalytic N2 fixation under N2 and Ar atmosphere over FMS catalyst. --------11

**Figure 15**. $^{1}H$ NMR spectra of photo catalytic reduction of dinitrogen to ammonia under 15N2 isotope labeled gas. (Conditions : DMSO-d6, 300 MHz. 1 JN-H = 71.4 Hz). ----------------------12

**Figure S16**. Recyclability of FMS catalyst. ----------------------------------------------------------13

**Figure S17**. TEM images of FMS catalyst: Before and after catalytic reaction. --------------------13

**Figure S18**. XPS analysis of FMS catalyst after photocatalytic reaction. ---------------------------14

**Figure S19**. Possible mechanism of present photocatalytic N2 fixation on FMS catalyst.--------15

**Figure S20**. Hydrazine colorimetric assay. (a) calibration curve. (b) concentration of Hydrazine as a function of photo irradiation------------------------------------------------------------------------15

**Figure S21**. Photocatalytic nitrogen fixation by using different proton sources on FMS catalyst. -------------------------------------------------------------------------------------------------------16

**Figure S22**. Photocatalytic N2 fixation under Dark and Light condition.----------------------------16

S22

**S2. Supplementry tables**

**Table S1**. Photocatalytic nitrogen fixation is studied at room temperature by using various nanomaterials.------------------------------------------------------------------------------------------17

**Table S2.** Comparing ammonia quantum yields over biomimetic photocatalysts reported in recent literature. ------------------------------------------------------------------------------------------------18

**Table S3**. Recent literature on photocatalytic dinitrogen reduction to ammonia over biomimetic

catalysts-----------------------------------------------------------------------------------------------19

S4. **Supplementary references**------------------------------------------------------------------------21

## S1. Supplementary figures

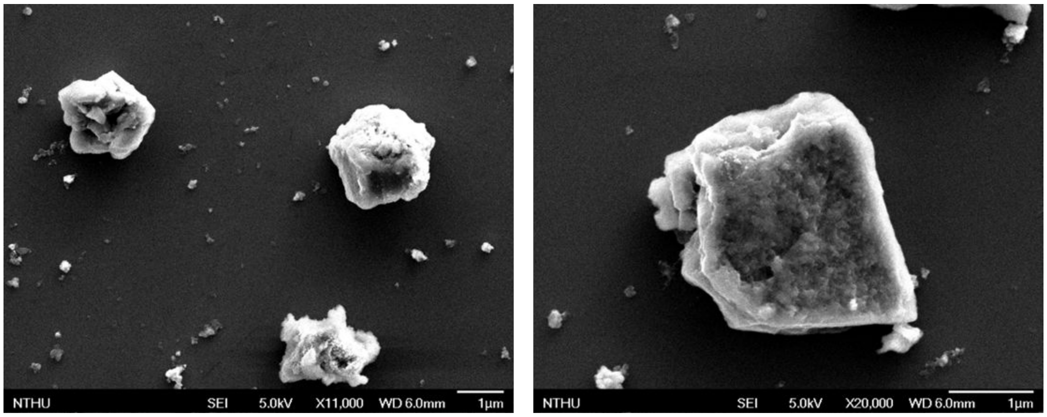


**Figure S1**. SEM images of FMS catalyst.

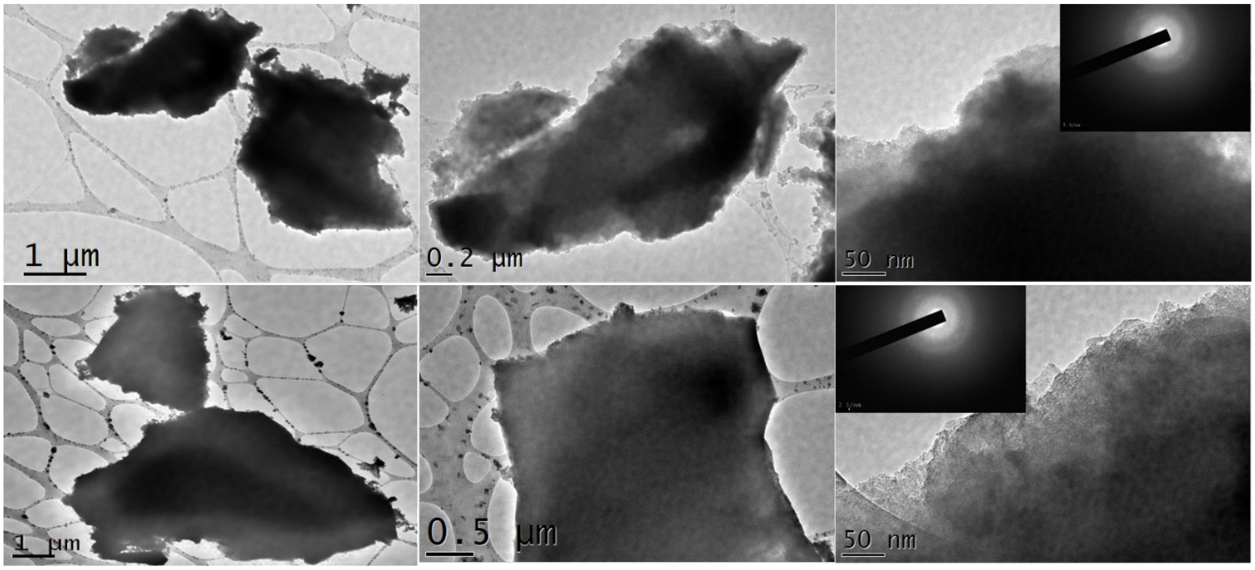


**Figure S2**. High and low resolution HRTEM images of FMS catalyst.

BET Surface Area Report

| | | |
|---|---|---|
| BET Surface Area: | 3.0946 ± | 0.0350 $m^2/g$ |
| Slope: | 1.381169 ± | 0.015765 |
| Y-Intercept: | 0.025560 ± | 0.002303 |
| C: | 55.036567 | |
| VM: | 0.710869 | $cm^3/g$ STP |
| Correlation Coefficient: | 9.998046e-01 | |
| Molecular Cross-section: | 0.1620 | $nm^2$ |

| Relative Pressure | Vol Adsorbed ($cm^3/g$ STP) | 1/ [VA*(Po/P - 1)] |
|---|---|---|
| 0.068964523 | 0.6078 | 0.121863 |
| 0.080458659 | 0.6510 | 0.134402 |
| 0.120429882 | 0.7057 | 0.194010 |
| 0.180156702 | 0.8046 | 0.273121 |
| 0.220202672 | 0.8555 | 0.330082 |

**Figure S3**. BET surface area analysis report.

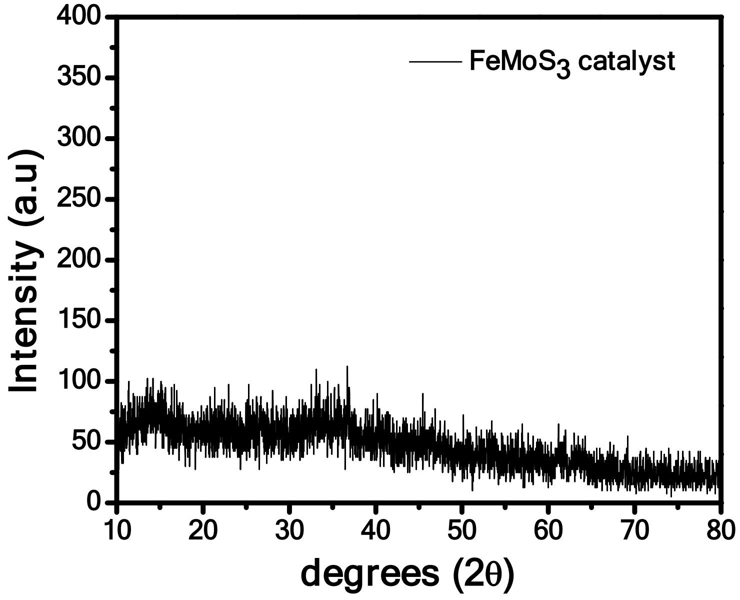


**Figure S4.** XRD spectra of $FeMoS_X$ microparticles .

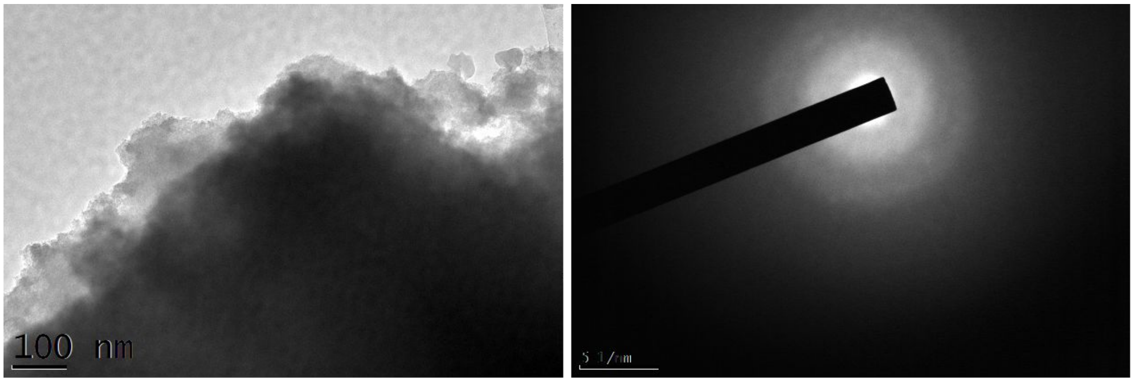


**Figure S5**. SAED pattern of $FeMoS_X$ particles

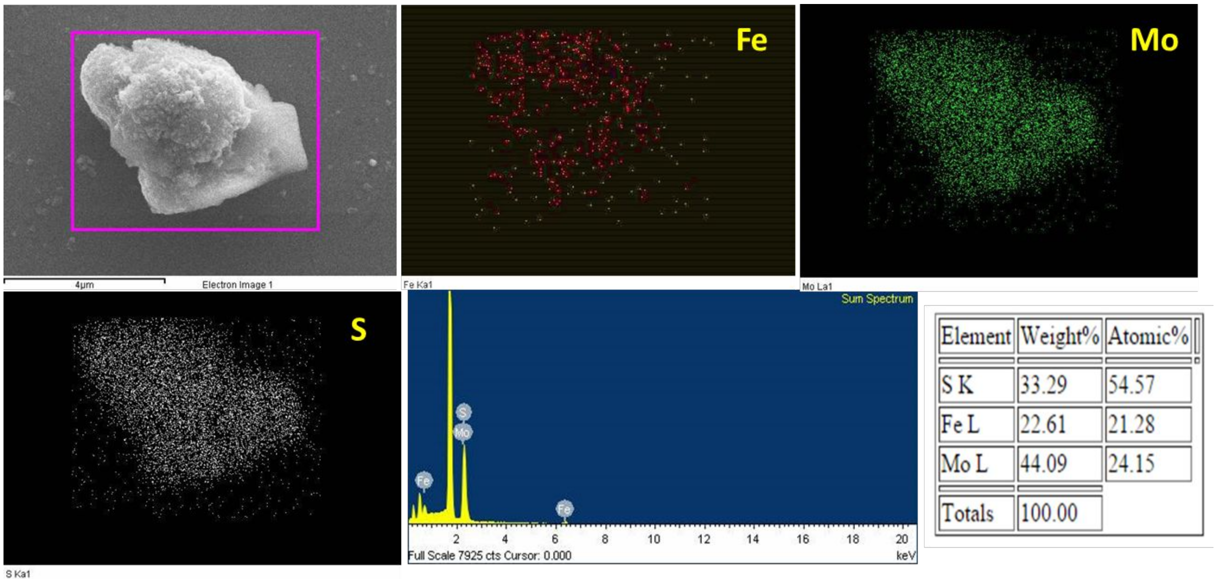


| Element | Weight% | Atomic% |
|---|---|---|
| S K | 33.29 | 54.57 |
| Fe L | 22.61 | 21.28 |
| Mo L | 44.09 | 24.15 |
| Totals | 100.00 | |

**Figure 6. EDX** Elemental composition analysis of $FeMoS_X$ microparticles.

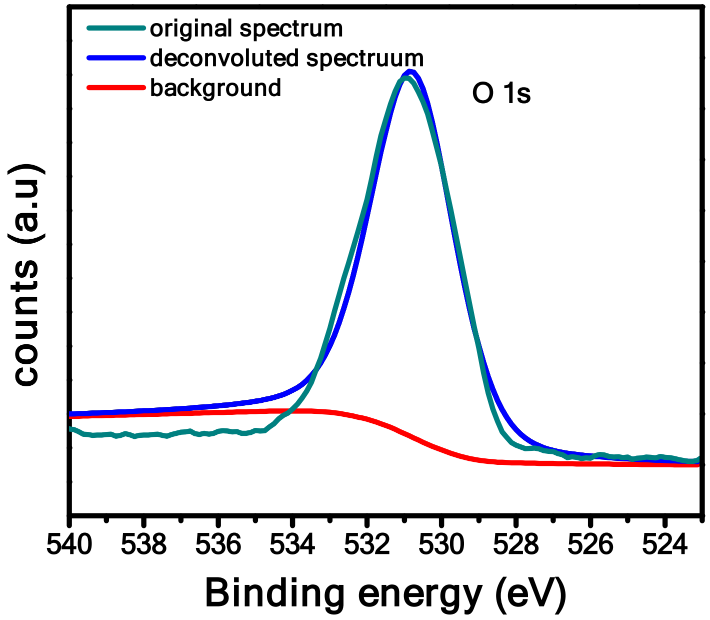


**Figure S7**. High reosolution XPS spectra of O 1s.

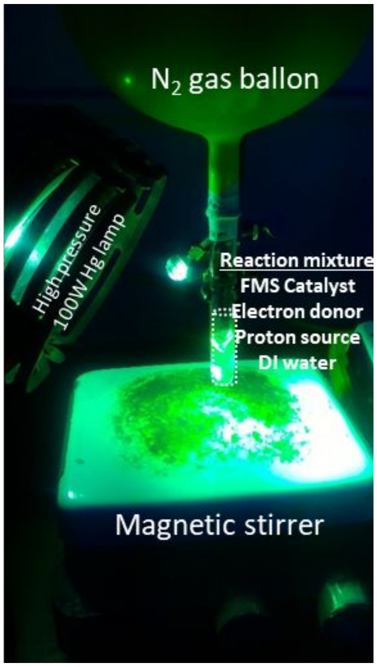


**Figure S8**. Photograph of the reaction setup for photochemical dinitrogen reduction reaction.

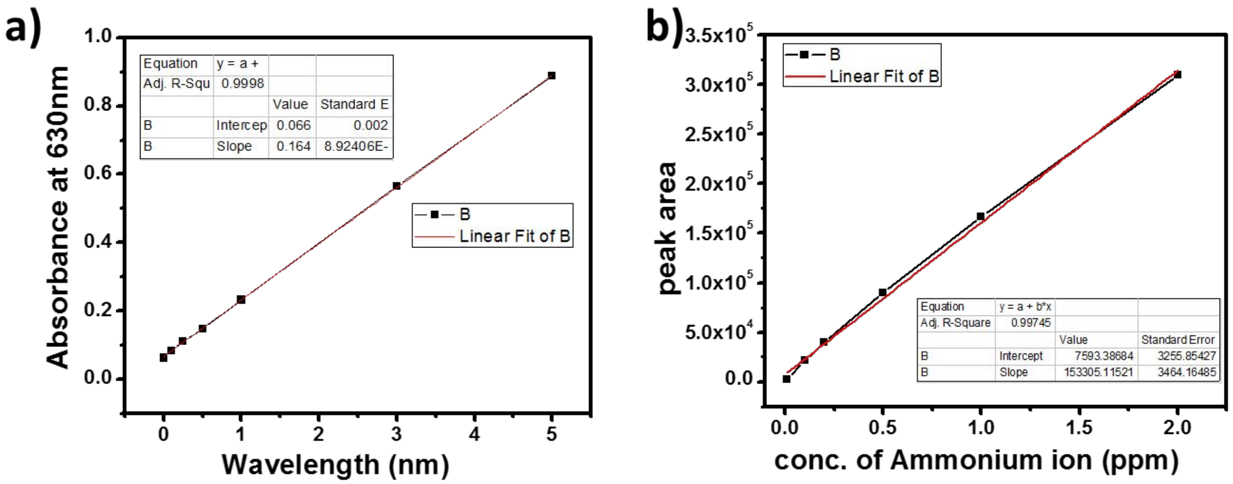


**Figure S9. standard calibration curves.** (a) calibration curve for Indophenol assay. (b) calibration curve for Ion chromatogram.

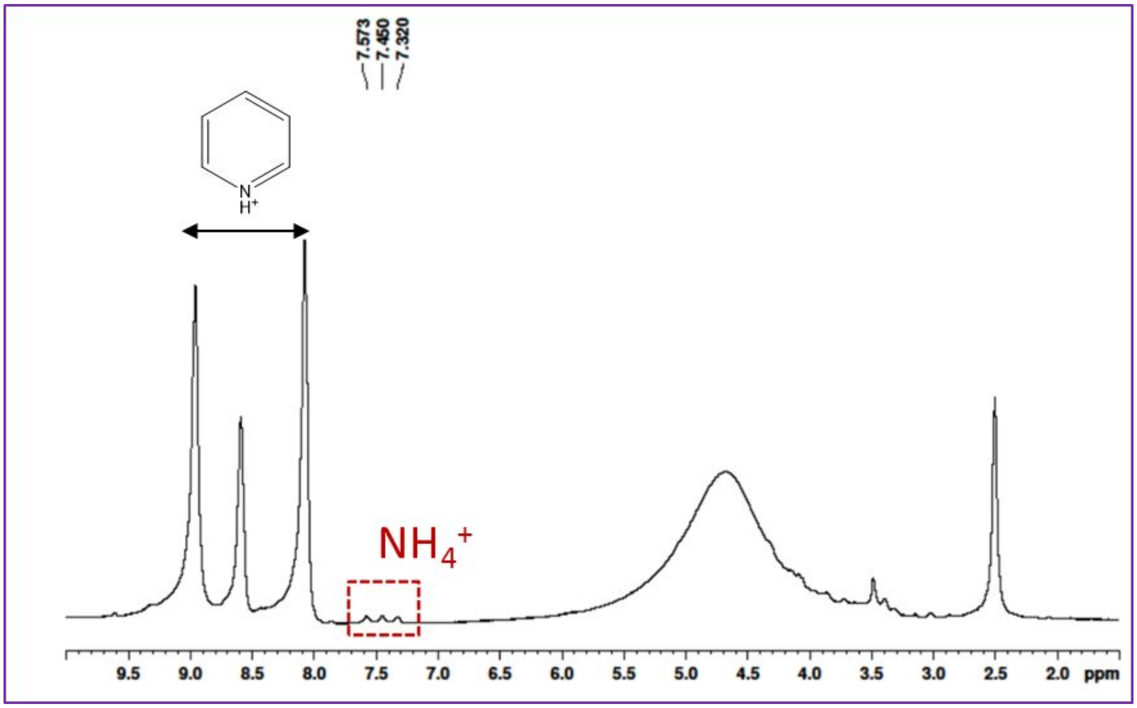


**Figure S10. $^{1}$H NMR spectra.** $^1$H NMR spectra of photo catalytic reduction of dinitrogen to ammonia at standard experimental condition. Conditions : DMSO-$d_6$, 300 MHz. $^1J_{N\text{-}H}$ = 49.2 Hz.

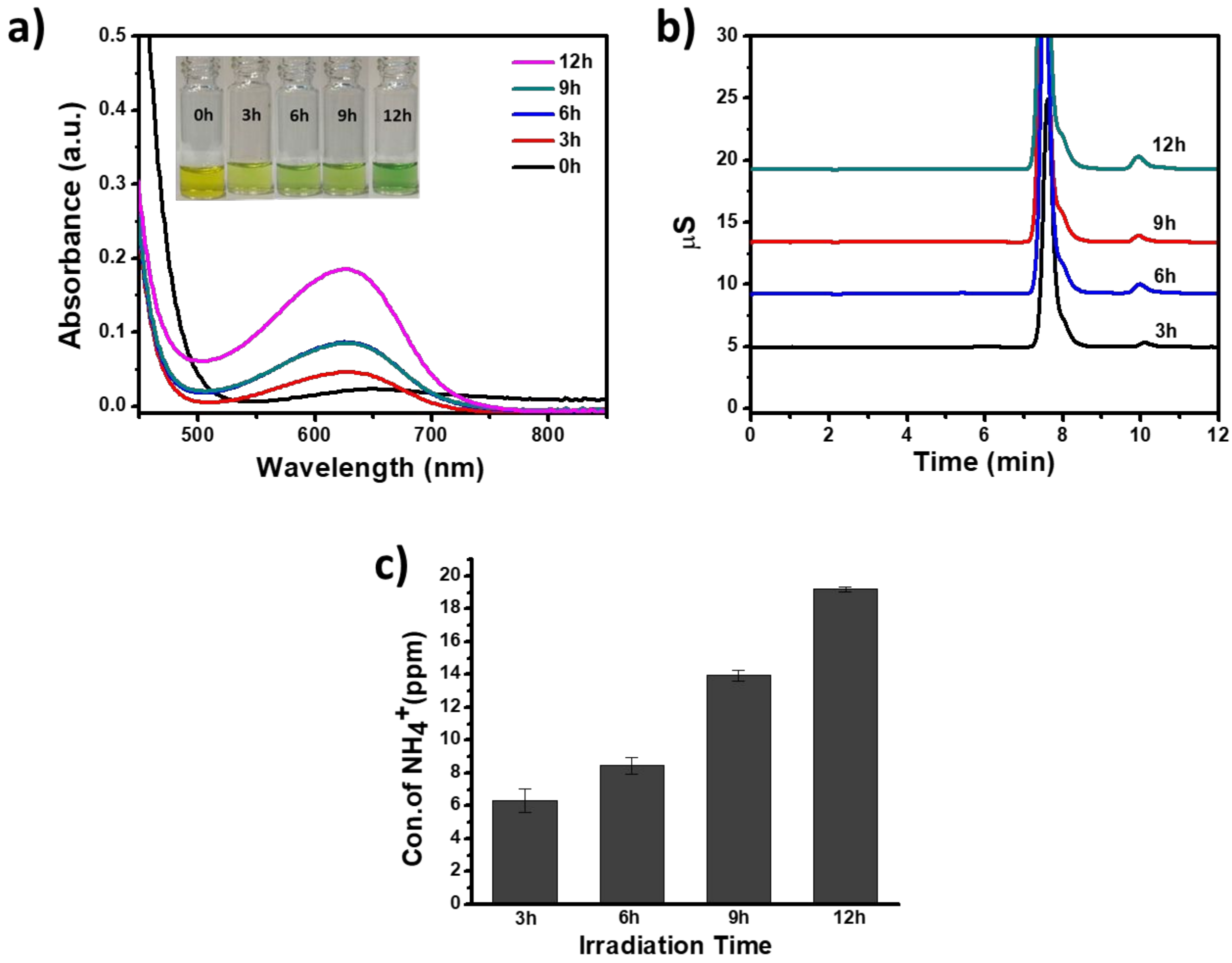


**Figure S11.** Photocatalytic dinitrogen reduction by using FMS-5h catalyst. a) Indophenol qualitative assay. b) Ion-chromatogram quantitative assay. c) kinetic plot of ammonia generation during the photoirradiation.

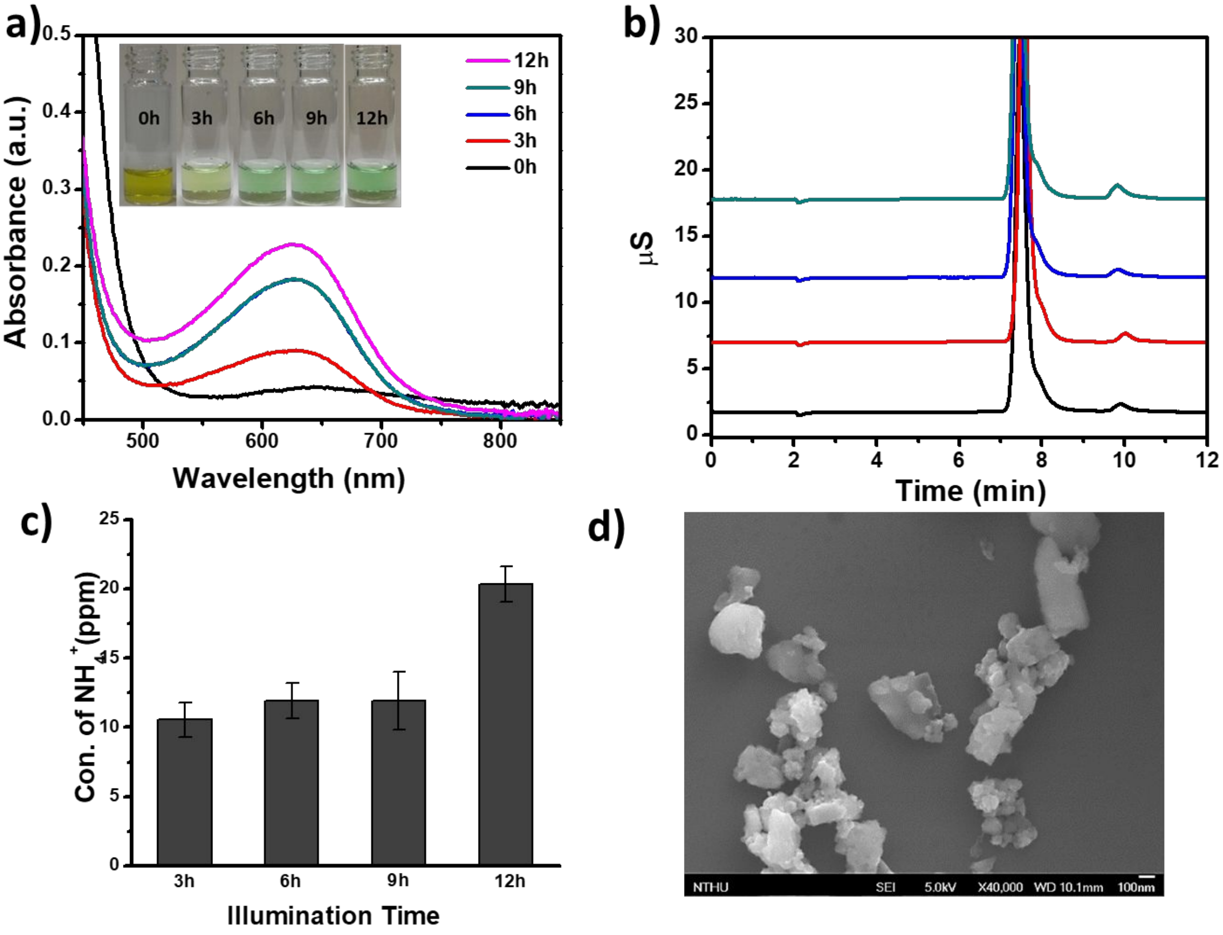


**Figure S12.** Photocatalytic dinitrogen reduction by using FMS-15h catalyst. a) Indophenol qualitative assay. b) Ion-chromatogram quantitative assay. c) kinetic plot of ammonia generation during the photoirradiation. d) SEM image of the FMS-15h catalyst.

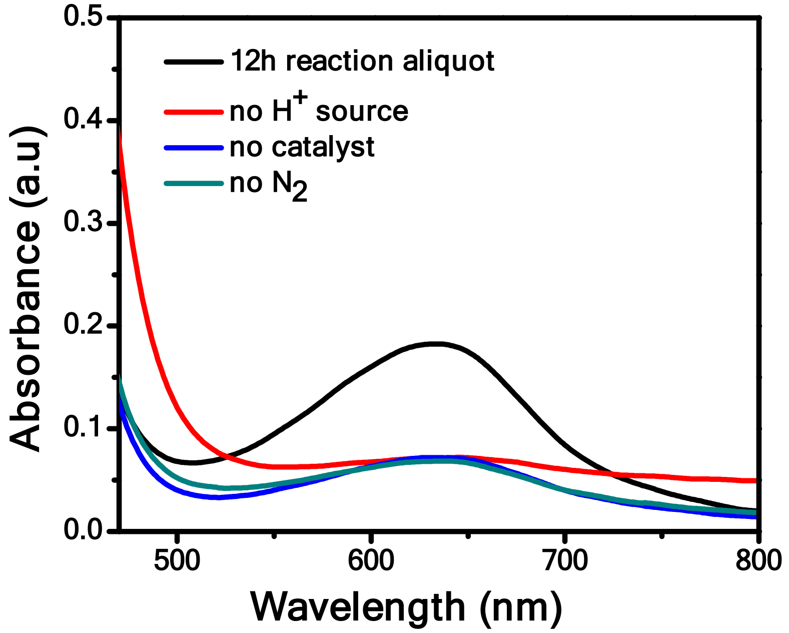


**Figure S13.** Representative UV-Vis of indophenol assays of 12h aliquots obtained during photocatalytic dinitrogen reduction at different conditions.

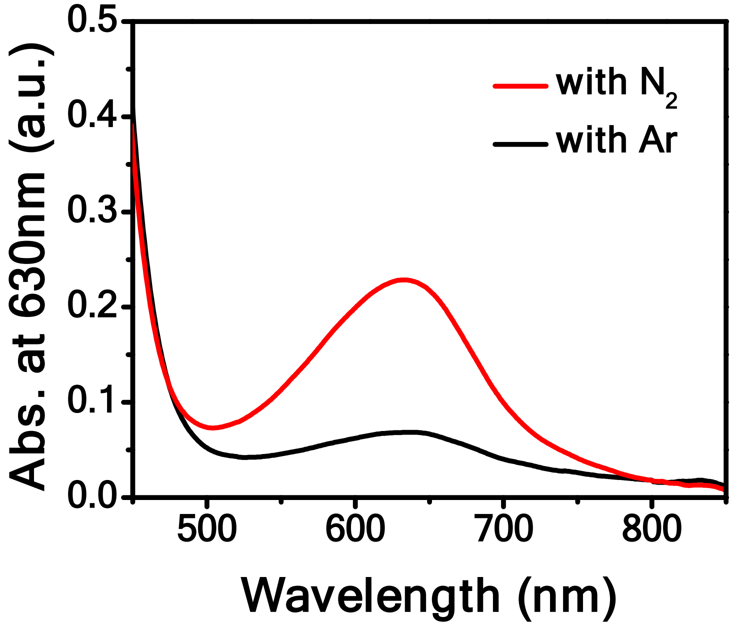


**Figure S14.** Photocatalytic $N_2$ fixation under $N_2$ and Ar atmosphere over FMS catalyst.

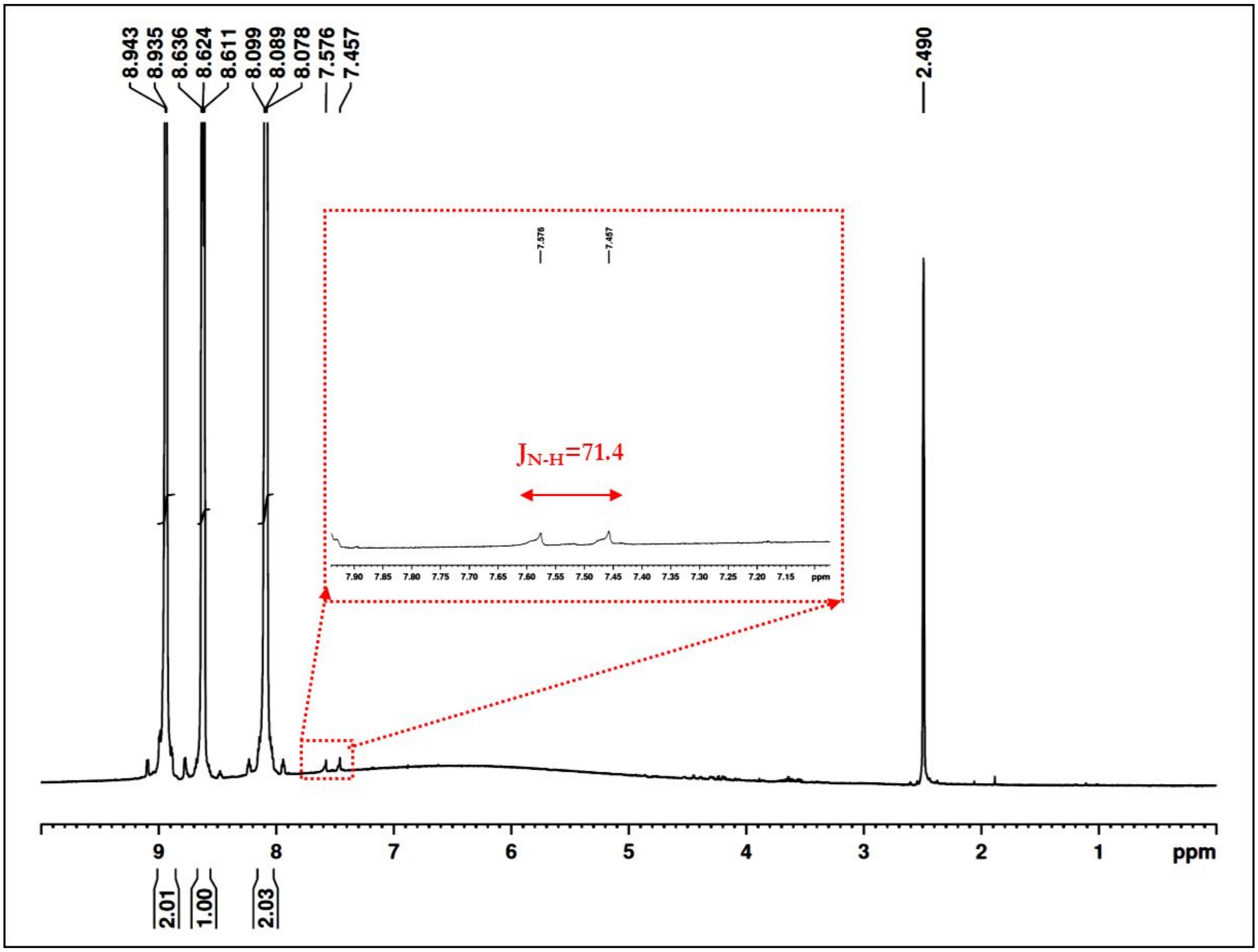


**Figure 15**. $^1$H NMR spectra of photo catalytic reduction of dinitrogen to ammonia under $^{15}N_2$ isotope labeled gas. (Conditions : DMSO-$d_6$, 300 MHz. $^1J_{N\text{-}H}$ = 71.4 Hz).

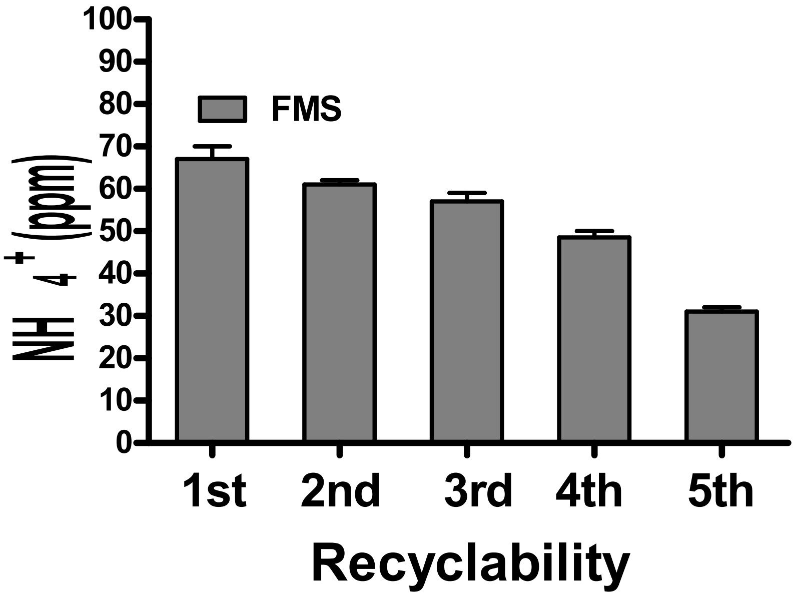


**Figure S16**. Recyclability of FMS catalyst.

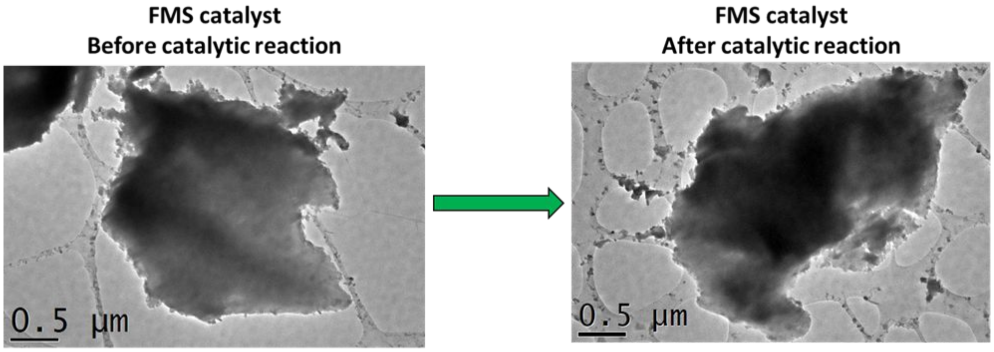


**Figure S17**. TEM images of FMS catalyst: before and after photocatalytic reaction.

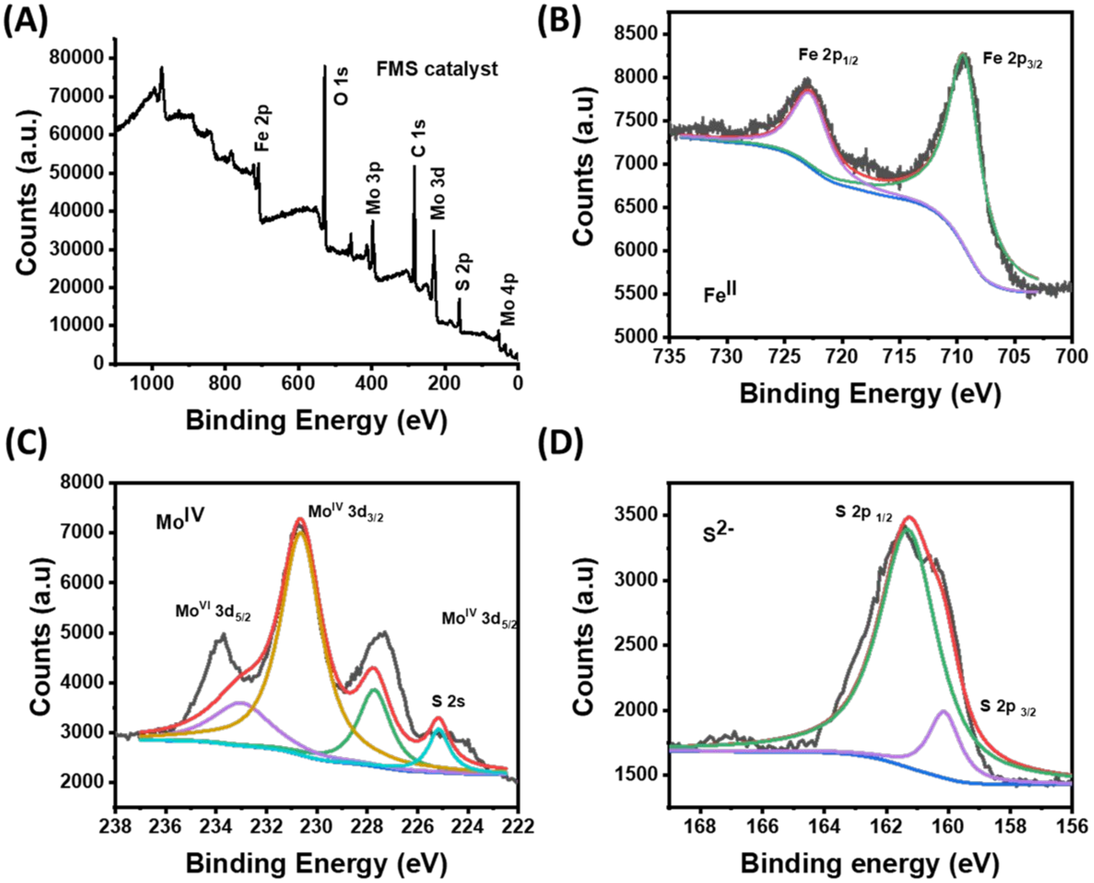


**Figure S18.** XPS analysis of FMS catalyst after photocatalytic reaction (A) in a wide range covering all Fe, O, Mo, S elements, (B) a magnified range between 735~700 eV for the Fe element, (C) a magnified range between 238~222 eV for various Mo oxidation states, and (D) 167~156 eV for the S element.

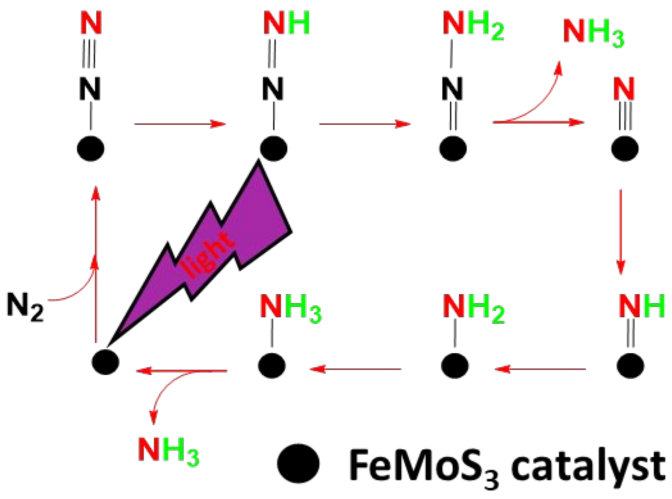


**Figure S19**. **P**ossible mechanism of present photocatalytic $N_2$ fixation on FMS catalyst.

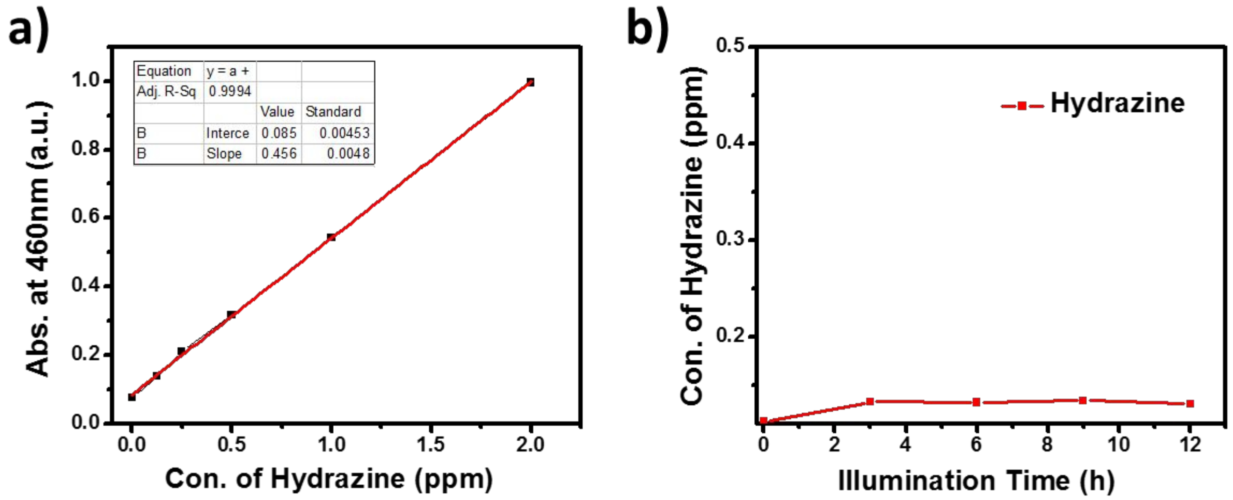


**Figure S20. Hydrazine colorimetric assay.** (a) calibration curve. (b) concentration of Hydrazine as a function of photo irradiation

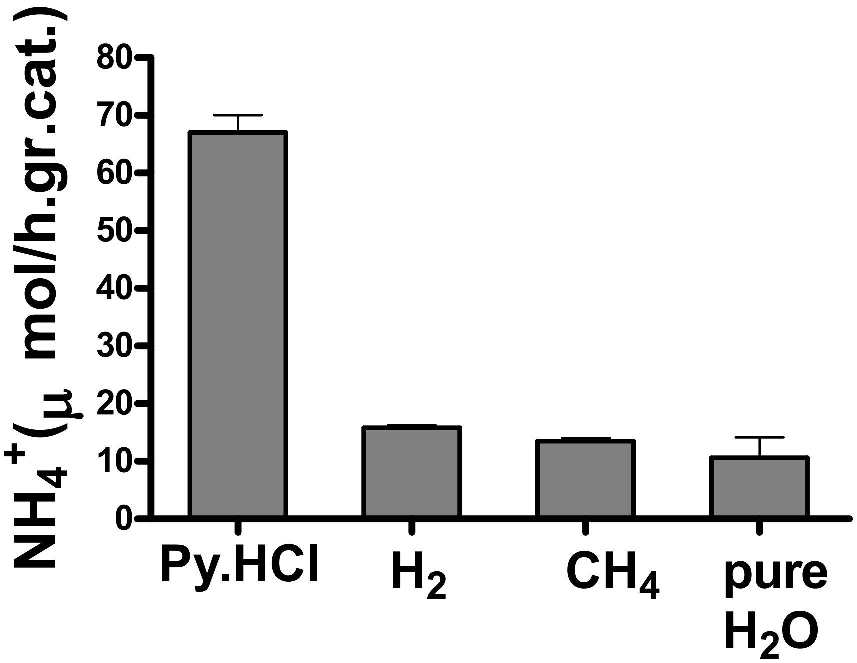


**Figure S21**. Photocatalytic nitrogen fixation by using different proton sources on FMS catalyst.

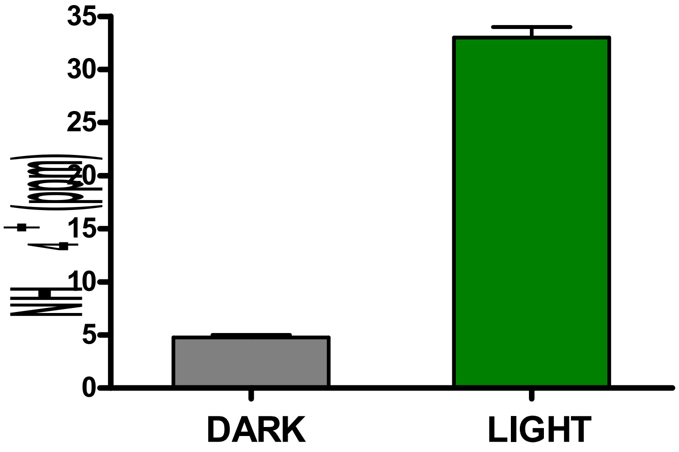


**Figure S22**. Photocatalytic $N_2$ fixation under Dark and Light condition.

**Table S1. Photocatalytic dinitrogen fixation was studied at room temperature by using various nanomaterials.**

| Catalyst | Reaction condition | Light source | Ammonia Production rate ($\mu mol \cdot h^{-1} g^{-1}$) | Catalyst synthesis protocol reference |
|---|---|---|---|---|
| FMS | $H_2O$, Proton source, electron donor, RT, 12h | 100 w Hg Lamp (full spectrum) | 64.005 | This work |
| FMS-5h | ,, | ,, | 94.90 | This work |
| FMS-15h | ,, | ,, | 99.79 | This work |
| $Fe_2O_3$ NPs | ,, | ,, | 51.970 | S1 |
| NiO NPs | ,, | ,, | 48.73 | S2 |
| $Gd_2O_3$ SNPs | ,, | ,, | 47.18 | S3 |
| ZnO NPs | ,, | ,, | 17.71 | S4 |
| Ag NPs | ,, | ,, | 17.10 | S5 |
| Co NPs | ,, | ,, | 12.10 | S6 |
| Au NPs | ,, | ,, | 11.79 | S7 |
| $Sm_2O_3$ SNPs | ,, | ,, | 10.69 | S3 |
| $Eu_2O_3$ SNPs | ,, | ,, | 10.30 | S3 |
| $WS_2$ NSs | ,, | ,, | 9.26 | S8 |
| $MoS_2$ NSs | ,, | ,, | 7.211 | S8 |

**Table S2. Comparing ammonia quantum yields over biomimetic photocatalysts reported in recent literature.**

| Catalyst and Approach | condition | Reaction time (h) | Ammonia (in ppm) | Absolute yield (µmol/h. gr. of catalyst) | QE* (%) | Ref. |
|---|---|---|---|---|---|---|
| **$FeMoS_3$ microparticles** | **100W high pressure Hg lamp, $H_2O$, proton source, $e^-$ donor** | **12** | **13.056** | **64.00 ± 5.0** | **0.013** | **Present work** |
| **FMS-5h** | | | **19.19** | **94.1 ± 1.0** | **0.027** | |
| **FMS-15h** | | | **20.35** | **99.72 ± 6.0** | **0.028** | |
| FMZ-0.05 | 100W high pressure Hg lamp, H2O, proton source, e- donor | 12 | | | 0.92 | S9 |
| FeMoS-chalcogels | 150W Xe lamp, $H_2O$, proton source, $e^-$ donor | 48 | 5.3 | 20.1 | Not mentioned (~ <0.02) | S10 |
| FeMoSFeS-SnS chalcogel | 150W Xe lamp, $H_2O$, proton source, $e^-$ donor | 48 | 10.5 | -<br>- | Not mentioned (~ <0.02) | S11 |
| FeS-SnS chalcogel | | 48 | 16 | | | |

*Other factors that effectively influence the yield calculation such as light intensity, yield physical shape (i.e., solid, gas), weight of semiconductor and physical shape of the catalyst, are all crucial when comparing the yield in different catalyst.

**Table S3. Comparing ammonia yields over reported photocatalysts in recent literature.**

| Catalyst | Ammonia Production rate | *QE (%) | Ref. |
|---|---|---|---|
| BiO | 1226 $\mu mol \cdot h^{-1} g^{-1}$ | - | S12 |
| p-$BiOBr$/n-$Bi_2MoO_6$ Z-scheme hetero-nanofibers | 911.6 $\mu mol\ g^{-1}\ h^{-1}\ L^{-1}$ | - | S13 |
| AgPt–$TiO_2$ | 38.4 $\mu mol\ g^{-1}\ h^{-1}$ | - | S14 |
| Ru Modified g-$C_3N_4$ nanosheets | 2.627 $mg/h/g_{cat}$ | - | S15 |
| $AgCl$/δ-$Bi_2O_3$ nanosheets | 606 $\mu mol \cdot h^{-1}\ g^{-1}$ | - | S16 |
| Ultrathin $Mo_{1-x}W_xS_2$ nanosheets | 111 $\mu mol\ g_{cat}^{-1}\ h^{-1}$ | - | S17 |
| Mg1.1$Al_{0.3}Fe_{0.2}O_{1.7}$ | 417 $\mu mol\ g^{-1}\ h^{-1}\ L^{-1}$ | - | S18 |
| $TiO_2/SrTiO_3$/g-$C_3N_4$ nanofibers | 2192 $\mu mol\ g^{-1}\ h^{-1}\ L^{-1}$ | - | S19 |
| $Cu^+$-doped g-$C_3N_4$ | 489 $\mu mol\ g^{-1}\ h^{-1}\ L^{-1}$ | - | S20 |

| | | | |
|---|---|---|---|
| $Au/TiO_2$ | 130 μmol·$h^{-1}g^{-1}$ | 0.82 | S21 |
| BiOBr | 102 μmol·$h^{-1}g^{-1}$ | 0.23 | S22 |
| $Bi_5O_7Br$ | | 2.3 | S23 |
| $TiO_2$ | | - | S24 |
| Cu-doped $TiO_2$ | 78.9 μmol·$h^{-1}g^{-1}$ | 0.08 @600 nm and 0.05%@700 nm | S25 |
| single atom Ru decorated $TiO_2$ nanosheets | 56.3 μg/h/$g_{cat}$ | - | S26 |
| surface oxygen vacancies modified micro-nanosheet structure $Bi_2O_2CO_3$ (namely BOC/OV) | 1178 μmol·$L^{-1}$·$g^{-1}$·$h^{-1}$ | - | S27 |
| Ag/AgI-δ-$Bi_2O_3$ nanocomposites | 420 μmol $L^{-1}$ $g^{-1}$ $h^{-1}$ | - | S28 |
| g-$C_3N_4$ | 1.2 mmol·$h^{-1}g^{-1}$ | -- | S29 |
| g-$C_3N_4$-nitrogen vacancies | 333 μmol·g-1·h-1·L-1 | - | S30 |
| $Mo_{0.1}Ni_{0.1}Cd_{0.8}S$ | 3.2 mg.$L^{-1}h^{-1}$ | - | S31 |

**Supporting references:**


S1. Thangudu, S.; Tsai, C. Y.; Lin, W. C.; Su, C. H., Modified gefitinib conjugated Fe(3)O(4) NPs for improved delivery of chemo drugs following an image-guided mechanistic study of inner vs. outer tumor uptake for the treatment of non-small cell lung cancer. *Front Bioeng Biotechnol* **2023,** *11*, 1272492.

S2. Alvarado, J. A.; Maldonado, A.; Juarez, H.; Pacio, M., Synthesis of Colloidal ZnO Nanoparticles and Deposit of Thin Films by Spin Coating Technique. *Journal of Nanomaterials* **2013,** *2013*, 903191.

S3. Rajagopal, S.; Thangudu, S.; Hwang, K. C., Synthesis of high yield, crystalline and thermally stable rare earth (Sm, Eu, Gd) oxide square nanoplates for near-infrared light activatable photocatalysis. *Catalysis Science & Technology* **2023,** *13* (12), 3701-3708.

S4. Hashem, M.; Saion, E.; Al-Hada, N. M.; Kamari, H. M.; Shaari, A. H.; Talib, Z. A.; Paiman, S. B.; Kamarudeen, M. A., Fabrication and characterization of semiconductor nickel oxide (NiO) nanoparticles manufactured using a facile thermal treatment. *Results in Physics* **2016,** *6*, 1024-1030.

S5. Thangudu, S.; Kulkarni, S. S.; Vankayala, R.; Chiang, C.-S.; Hwang, K. C., Photosensitized reactive chlorine species-mediated therapeutic destruction of drug-resistant bacteria using plasmonic core–shell Ag@AgCl nanocubes as an external nanomedicine. *Nanoscale* **2020,** *12* (24), 12970-12984.

S6. Janjua, M. R. S. A., Synthesis of Co3O4 Nano Aggregates by Co-precipitation Method and its Catalytic and Fuel Additive Applications. *Open Chemistry* **2019,** *17* (1), 865-873.

S7. Rajagopal, S.; Thangudu, S.; Hwang, K. C., A high-index facet gold 12 tip nanostar for an improved electrocatalytic alcohol oxidation reaction with superior CO tolerance. *Nanoscale* **2023,** *15* (28), 11963-11971.

S8. Thangudu, S.; Lee, M. T.; Rtimi, S., Tandem Synthesis of High Yield MoS2 Nanosheets and Enzyme Peroxidase Mimicking Properties. *Catalysts* **2020,** *10* (9), 1009.

S9. Thangudu, S.; Wu, C.-H.; Lee, C.-H.; Hwang, K. C., Enhanced Photofixation of Dinitrogen to Ammonia over a Biomimetic Metal (Fe,Mo)-Doped Mesoporous MCM-41 Zeolite Catalyst under Ambient Conditions. *ACS Sustainable Chemistry & Engineering* **2021,** *9* (26), 8748-8758.

S10. Banerjee, A.; Yuhas, B. D.; Margulies, E. A.; Zhang, Y.; Shim, Y.; Wasielewski, M. R.; Kanatzidis, M. G., Photochemical Nitrogen Conversion to Ammonia in Ambient Conditions with FeMoS-Chalcogels. *Journal of the American Chemical Society* **2015,** *137* (5), 2030-2034.
S11. Liu, J.; Kelley, M. S.; Wu, W.; Banerjee, A.; Douvalis, A. P.; Wu, J.; Zhang, Y.; Schatz, G. C.; Kanatzidis, M. G., Nitrogenase-mimic iron-containing chalcogels for photochemical reduction of dinitrogen to ammonia. *Proceedings of the National Academy of Sciences* **2016,** *113* (20), 5530-5535.
S12. Sun, S.; An, Q.; Wang, W.; Zhang, L.; Liu, J.; Goddard Iii, W. A., Efficient photocatalytic reduction of dinitrogen to ammonia on bismuth monoxide quantum dots. *Journal of Materials Chemistry A* **2017,** *5* (1), 201-209.
S13. Wu, X.; Zhang, F.; Niu, L.; Liu, J.; Li, J.; Wang, D.; Fan, J.; Li, X.; Shao, C.; Li, X.; Liu, Y., Promoting photocatalytic nitrogen reduction for aqueous nitrogenous fertilizer from organic wastewater over p-BiOBr/n-Bi2MoO6 hetero-nanofibers. *Chemical Engineering Journal* **2023,** *470*, 144108.
S14. Bian, X.; Zhao, Y.; Zhang, S.; Li, D.; Shi, R.; Zhou, C.; Wu, L.-Z.; Zhang, T., Enhancing the Supply of Activated Hydrogen to Promote Photocatalytic Nitrogen Fixation. *ACS Materials Letters* **2021,** *3* (11), 1521-1527.
S15. Hao, D.; Ren, J.; Wang, Y.; Arandiyan, H.; Garbrecht, M.; Bai, X.; Shon, H. K.; Wei, W.; Ni, B.-J., A Green Synthesis of Ru Modified g-C<sub>3</sub>N<sub>4</sub> Nanosheets for Enhanced Photocatalytic Ammonia Synthesis. *Energy Material Advances* **2021,** *2021*.
S16. Gao, X.; Shang, Y.; Liu, L.; Fu, F., Chemisorption-enhanced photocatalytic nitrogen fixation via 2D ultrathin p–n heterojunction AgCl/δ-Bi2O3 nanosheets. *Journal of Catalysis* **2019,** *371*, 71-80.
S17. Qin, J.; Zhao, W.; Hu, X.; Li, J.; Ndokoye, P.; Liu, B., Exploring the N2 Adsorption and Activation Mechanisms over the 2H/1T Mixed-Phase Ultrathin Mo1–xWxS2 Nanosheets for Boosting N2 Photosynthesis. *ACS Applied Materials & Interfaces* **2021,** *13* (6), 7127-7134.
S18. Wang, Y.; Wei, W.; Li, M.; Hu, S.; Zhang, J.; Feng, R., In situ construction of Z-scheme g-C3N4/Mg1.1Al0.3Fe0.2O1.7 nanorod heterostructures with high N2 photofixation ability under visible light. *RSC Advances* **2017,** *7* (29), 18099-18107.

S19. Tao, R.; Li, X.; Li, X.; Shao, C.; Liu, Y., $TiO_2/SrTiO_3/g-C_3N_4$ ternary heterojunction nanofibers: gradient energy band, cascade charge transfer, enhanced photocatalytic hydrogen evolution, and nitrogen fixation. *Nanoscale* **2020,** *12* (15), 8320-8329.
S20. Hu, S.; Qu, X.; Bai, J.; Li, P.; Li, Q.; Wang, F.; Song, L., Effect of Cu(I)–N Active Sites on the $N_2$ Photofixation Ability over Flowerlike Copper-Doped $g-C_3N_4$ Prepared via a Novel Molten Salt-Assisted Microwave Process: The Experimental and Density Functional Theory Simulation Analysis. *ACS Sustainable Chemistry & Engineering* **2017,** *5* (8), 6863-6872.
S21. Yang, J.; Guo, Y.; Jiang, R.; Qin, F.; Zhang, H.; Lu, W.; Wang, J.; Yu, J. C., High-Efficiency "Working-in-Tandem" Nitrogen Photofixation Achieved by Assembling Plasmonic Gold Nanocrystals on Ultrathin Titania Nanosheets. *Journal of the American Chemical Society* **2018,** *140* (27), 8497-8508.
S22. Li, H.; Shang, J.; Ai, Z.; Zhang, L., Efficient Visible Light Nitrogen Fixation with BiOBr Nanosheets of Oxygen Vacancies on the Exposed {001} Facets. *Journal of the American Chemical Society* **2015,** *137* (19), 6393-6399.
S23. Wang, S.; Hai, X.; Ding, X.; Chang, K.; Xiang, Y.; Meng, X.; Yang, Z.; Chen, H.; Ye, J., Light-Switchable Oxygen Vacancies in Ultrafine $Bi_5O_7Br$ Nanotubes for Boosting Solar-Driven Nitrogen Fixation in Pure Water. *Advanced Materials* **2017,** *29* (31), 1701774.
S24. Hirakawa, H.; Hashimoto, M.; Shiraishi, Y.; Hirai, T., Photocatalytic Conversion of Nitrogen to Ammonia with Water on Surface Oxygen Vacancies of Titanium Dioxide. *Journal of the American Chemical Society* **2017,** *139* (31), 10929-10936.
S25. Zhao, Y.; Zhao, Y.; Shi, R.; Wang, B.; Waterhouse, G. I. N.; Wu, L.-Z.; Tung, C.-H.; Zhang, T., Tuning Oxygen Vacancies in Ultrathin $TiO_2$ Nanosheets to Boost Photocatalytic Nitrogen Fixation up to 700 nm. *Advanced Materials* **2019,** *31* (16), 1806482.
S26. Liu, S.; Wang, Y.; Wang, S.; You, M.; Hong, S.; Wu, T.-S.; Soo, Y.-L.; Zhao, Z.; Jiang, G.; Jieshan, Q.; Wang, B.; Sun, Z., Photocatalytic Fixation of Nitrogen to Ammonia by Single Ru Atom Decorated $TiO_2$ Nanosheets. *ACS Sustainable Chemistry & Engineering* **2019,** *7* (7), 6813-6820.
S27. Feng, Y.; Zhang, Z.; Zhao, K.; Lin, S.; Li, H.; Gao, X., Photocatalytic nitrogen fixation: Oxygen vacancy modified novel micro-nanosheet structure $Bi_2O_2CO_3$ with band gap engineering. *Journal of Colloid and Interface Science* **2021,** *583*, 499-509.

S28. Gao, X.; Shang, Y.; Gao, K.; Fu, F., Plasmon Sensitized Heterojunction 2D Ultrathin Ag/AgI-δ-Bi(2)O(3) for Enhanced Photocatalytic Nitrogen Fixation. *Nanomaterials (Basel)* **2019,** *9* (5).
S29. Dong, G.; Ho, W.; Wang, C., Selective photocatalytic N2 fixation dependent on g-C3N4 induced by nitrogen vacancies. *Journal of Materials Chemistry A* **2015,** *3* (46), 23435-23441.
S30. Wu, G.; Gao, Y.; Zheng, B., Template-free method for synthesizing sponge-like graphitic carbon nitride with a large surface area and outstanding nitrogen photofixation ability induced by nitrogen vacancies. *Ceramics International* **2016,** *42* (6), 6985-6992.
S31. Cao, Y.; Hu, S.; Li, F.; Fan, Z.; Bai, J.; Lu, G.; Wang, Q., Photofixation of atmospheric nitrogen to ammonia with a novel ternary metal sulfide catalyst under visible light. *RSC Advances* **2016,** *6* (55), 49862-49867.
